# Traditional Heart-Healthy Diet and Medication Adherence in the Norton Sound Region: An 18-Month Telehealth Intervention

**DOI:** 10.3390/ijerph19169885

**Published:** 2022-08-11

**Authors:** Marily Oppezzo, Mariah Knox, Jordan Skan, Amy Chieng, Maria Crouch, Rachael C. Aikens, Neal L. Benowitz, Matthew Schnellbaecher, Judith J. Prochaska

**Affiliations:** 1 Stanford Prevention Research Center, Department of Medicine, Stanford University, Palo Alto, CA 94304, USA; 2 Cardiology Department, Alaska Native Tribal Health Consortium, Anchorage, AK 99508, USA; 3 Department of Psychiatry, Yale School of Medicine, New Haven, CT 06510, USA; 4 Biomedical Informatics, Stanford University, Stanford, CA 94305, USA; 5 Division of Cardiology, Department of Medicine, University of California, San Francisco, CA 94158, USA

**Keywords:** telehealth, cardiovascular disease, Alaska Native, dietary intake, medication adherence

## Abstract

Introduction. Innovations are needed for preventing cardiovascular disease (CVD) and for reaching diverse communities in remote regions. The current study reports on a telemedicine-delivered intervention promoting a traditional heart-healthy diet and medication adherence with Alaska Native men and women residing in the Norton Sound region of Alaska. Methods. Participants were 299 men and women with high blood pressure or high cholesterol smoking daily who were randomized to receive telemedicine-delivered counseling and printed materials on diet and medication adherence or on smoking and physical activity. Intervention contacts were at baseline and 3-, 6-, and 12-months follow-up, with a final assessment at 18 months. Nutrition outcomes were the ratio of heart-healthy foods and traditional heart-healthy foods relative to all foods reported on a 34-item food frequency questionnaire. Recent and typical adherence for heart medications were self-reported. Results. Intervention effects were significant for the heart-healthy foods ratio at 6 months only (*p* = 0.014) and significant for the traditional heart-healthy foods ratio at 6 months only for those aged 47+ (*p* = 0.031). For recent and typical medication adherence, there were no significant group differences by time. Discussion. In a remote region of Alaska, telemedicine proved feasible and acceptable for engaging Alaska Native men and women in counseling on CVD risk behaviors. The findings indicate that more touchpoints may be necessary to impart comprehensive lasting change in heart-healthy eating patterns. Medication adherence group differences were not significant; however, medication adherence was high overall.

## 1. Introduction

Cardiovascular disease (CVD) is the second leading cause of death for Alaska Native people, with a mortality rate of 183.3 per 100,000, significantly higher than among Alaska non-Native people (120.8 per 100,000) [1]. The CVD mortality rate is even higher for Alaska Native people in the Norton Sound Region of Alaska (211.8 per 100,000) [2].

An estimated 79% of American Indian and Alaska Native adults have at least one CVD risk factor, and 46% have two or more [3]. Interventions that address multiple risk behaviors for change offer a comprehensive approach to reducing disease risk [4]. For the Norton Sound region, treatment strategies must be culturally targeted and remotely accessible.

In Alaska, declines in traditional food consumption have shifted toward more shelf-stable, highly processed, store-bought foods associated with a higher risk of CVD [5,6,7,8]. In more rural and removed Alaska Native communities, there are challenges to obtaining whole, fresh, and minimally processed foods, as many commercial and Western foods must be flown in on small planes sometimes without refrigeration [9,10]. Therefore, fresh, whole foods are often more expensive and less available. The traditional diet of Alaska Native people, however, comes from the land, and is high in marine sources of omega-three fatty acids (e.g., salmon and seal), which are linked to cardiovascular health [11,12,13,14]. Omega-3 fatty acids decrease CVD risk through a number of different mechanisms [15], and recently, a cross-sectional study demonstrated significant inverse relationships between habitual omega 3 intake levels in Yup’ik Alaska Native adults (via a validated blood biomarker) and three systemic inflammatory biomarkers [16]. Adherence to traditional dietary practices also correlate with the connection to one’s culture and community, which, in turn, have been associated with greater mental well-being and greater quality of life [7,17,18,19].

Lipid-lowering and antihypertensive medications also reduce the risk of CVD, but adherence can be poor. In the general population, about half of patients achieve adherence to heart medications [20,21]. For Alaska Native people, federal funding and other insurances provide coverage of many heart medications, with specifics varying by region. Barriers to medication adherence, however, extend beyond cost [22] such as: preferring lifestyle changes over medication; disliking medication; having trouble remembering to take medication [21].

Intervention trials that tackle multiple risk behaviors can be an impactful and ethical way to address a broader profile of participants’ disease risk. However, the literature documents mixed effects. One meta-analysis noted only modest risk factor changes [23], while another review found success intervening on multiple risk behaviors when there was a common disease state focus [4]. More research is needed to examine interventions on multi-behavioral risk factors, and specifically in understudied, high-risk communities [4,24,25].

The Healing and Empowering Alaskan Lives Toward Healthy-Hearts (HEALTHH) was designed in response to the National Heart, Lung, and Blood Institute’s request for applications for indigenous-health-focused research on multiple health behavior changes for secondary prevention of CVD [26]. The HEALTHH study aimed to test two culturally tailored, telemedicine-delivered interventions, based in the Transtheoretical Model of behavior change [27,28], each focused on changing two CVD risk behaviors to improve heart health. One condition promoted traditional, heart-healthy food intake and adherence to cholesterol-lowering and antihypertensive medications; the other condition promoted tobacco cessation and regular physical activity (outcomes to be reported elsewhere). The current paper compares changes in dietary intake and medication adherence between the two study conditions. We hypothesized that those randomly assigned to the nutrition and medication adherence condition would increase the self-reported intake of traditional heart-healthy foods; increase heart medication adherence, if prescribed medication.

## 2. Materials and Methods

### 2.1. Sample

Participants were 299 men and women aged 19 or older, identifying as Alaska Native, residing in 1 of 16 communities in Alaska’s Norton Sound region, with population sizes ranging from 150 to 3598 (which is the population size of Nome). Approximately 76% of the region’s population is of AN heritage, with the largest representation being Inupiaq and Yup’ik. Participants were recruited between 2015 and 2018 through comprehensive, community outreach and community engagement [29]. Additional inclusion criteria were: English-literate; smoking 5 or more cigarettes per day; having high blood pressure (systolic/diastolic BP ≥ 140 mmHg/90 mmHg) or high cholesterol (LDL ≥ 160) or currently prescribed antihypertensives or cholesterol-lowering medication [30]. Exclusion criteria were BMI > 50, being pregnant, or currently receiving tobacco cessation treatment.

**Figure 1:**
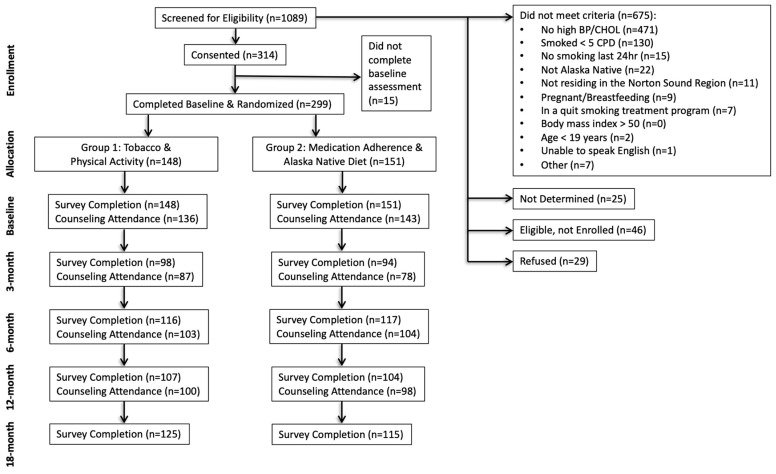
CONSORT diagram. Recruitment and retention by condition at each study check-point.

Participant enrollment and allocation are summarized in the CONSORT diagram (Figure 1). Leading reasons for study exclusion were not having hypertension or high cholesterol or smoking < 5 cigarettes daily.

### 2.2. Study Design

Following baseline assessment, a computer-generated, stratified random assignment program individually randomized participants based on their village size (Nome vs. other), cigarettes per day (cut-point of 8 [31]), and stage of change for quitting smoking. Randomization was to one of two study conditions: nutrition and medication adherence or tobacco cessation and physical activity. To avoid cross-group contamination, participants living in the same household were randomized as a pair to the same condition. Counseling sessions were provided after randomization at baseline, 3-, 6-, and 12-months follow-up, with assessments at baseline, 3-, 6-, 12-, and 18-months follow-up. The protocol has been described in detail [30]. Institutional review board (IRB) approvals were obtained from Stanford University; the University of California, San Francisco; the Alaska Area IRB; the Alaska Native Tribal Health Consortium Board and its manuscript and proposal review committee; the Norton Sound Board of Directors and its Research Ethics Review Board, the latter of which has closely guided the HEALTHH project.

### 2.3. Study Interventions

Given the remote location, the study’s computer-driven interventions were delivered via video telemedicine (Vidyo, a HIPAA-compliant video telemedicine platform [32]) to participants in their local community clinics by study health coaches. The study health coaches had college degrees in psychology, public health, or related fields. All study team members received extensive on-boarding training in the following: motivational interviewing; Alaska Native indigenous cultures; the history of research ethics violations with the Alaska Native peoples; dietary, tobacco, physical activity, and medication adherence guidelines; stage-tailored interventions; the use of the Transtheoretical Model-guided computer-counseling program [29,30]. The study health coaches provided counseling to participants in both conditions guided by a computer program that tailored counseling messages and material to the participant and was based on the Transtheoretical Model of change [33]. Counseling promoted a heart-healthy Alaska Native diet and heart medication adherence or tobacco cessation and physical activity, depending on study randomization. Because counseling sessions were individualized based on the participant’s stage of change, contamination across conditions was minimized.

The intervention materials for each condition were directly informed by the research team’s prior fieldwork in Alaska and continued community partnership [31,34,35] and culturally targeted to reflect traditional Alaska Native values such as respect for elders, land, and family, and included photos of Alaska Native traditional foods, land, and people. The nutrition and medication adherence condition provided a cookbook featuring heart-healthy Alaska Native recipes [36] and a bag to organize medications. As produce is not widely available and is often expensive in many Alaskan regions, we adapted nutrition materials to focus on the promotion of traditional foods rich in heart-healthy omega-3 polyunsaturated fats from marine mammals and fish [5,13,37], rather than the USDA guidelines’ emphasis on increased produce intake. Counseling sessions and materials highlighted participants’ regular access to traditional foods through subsistence activities, food sharing, public eateries, and community celebrations/gatherings. The intervention materials were reviewed by team members of Alaska Native heritage; data safety monitoring board members of American Indian and Alaska Native heritage; the Norton Sound Research Ethics Review Board, comprised of tribal stakeholders; the Alaska Area Institutional Review Board. Feedback-informed enhancements and targeting of the intervention included photos, language and cultural terms, and Alaska Native stories. The tobacco cessation and physical activity condition received a pedometer and 12 weeks of combination nicotine replacement therapy. For this paper, the tobacco cessation and physical activity condition is referred to as the comparison condition. Table 1 compares the intervention treatment components.

### 2.4. Measures

Study measures were collected by interview in person or by phone.

#### 2.4.1. Dietary Intake

A 34-item food frequency questionnaire, adapted from a measure used in Alaska Native communities in the Southeast Region of Alaska [38], asked how often over the past 7 days participants ate various foods. Response options for each food item were: Did not eat it this week; Once this week; 2–3 times this week; 4–6 times this week; Once or twice each day; More than twice each day; Refused (i.e., declined to answer). Nine of the 34 foods were traditional to the Alaska Native diet, or local to the region and enjoyed by communities prior to Western state influences (e.g., wild berries, moose, and whale oil) [9]. These were selected in consultation with team members who were of Alaska Native origin to reflect the traditional diet in the Norton Sound region. Sixteen of the 34 foods were considered heart-healthy, as determined by a dietitian and in line with either the DASH (Dietary Approach to Stopping Hypertension) diet or research on the heart healthiness of foods native to the Alaskan region, and each was available in the regions of study; a subset of 7 foods were both traditional and heart-healthy (e.g., traditional greens, whale skin and fat, walrus soup, and wild berries) [7,39]. Nontraditional, nonhealthy foods that were assessed included foods like fruit juice, soda, fruit canned in syrup, and spam. The full nutrition measure can be found in the Supplementary Materials. To minimize participant burden and survey fatigue, the 34-item food questionnaire was not intended to be an all-inclusive list of foods consumed. Instead, the foods were selected with the team members of Alaska Native origin to serve as a proxy for traditional and heart-healthy choices in a subset of foods, and to provide a consistent measure of this proxy over time.

For analyses, interpretability, and to be consistent with the baseline paper analyses (under review), response options were converted to estimate the number of times consumed per week with recoding to the midpoint of the range when needed (e.g., once or twice each day was recoded as 10.5, the midpoint between 7 and 14 times in a week). Reports of consumption more than twice daily were recoded as 14, the minimum, to be conservative and not inflate the distribution of responses. The two primary outcomes were ratios reflecting the relative change in the relationship of healthy to nonhealthy foods assessed. The ratio of heart-healthy foods was calculated as the sum total of times per week that heart-healthy foods on the list were consumed divided by the sum total for all foods reported. The ratio of traditional heart-healthy foods was a subset of the heart-healthy foods that were traditional, summed, and divided by the sum total for all foods reported.

#### 2.4.2. Medication Adherence

Two questions assessed primary outcomes of recent (“Did you take all your medicine as prescribed yesterday?”, yes = adherent) and typical (“How often do you have difficulty remembering to take all your medications?”, never/rarely = adherent) adherence to heart medications [40]. Those who responded “don’t know” were categorized conservatively as nonadherent. Those who refused to answer were not included in the analyses.

Measures of secondary outcomes were stages of change for heart medication adherence (i.e., for cholesterol and/or hypertension) asked of participants responding yes to the question, “has a doctor ever recommended that you take medication for (lowering cholesterol/high blood pressure) [33]?” The staging measures defined adherence as: “Consistently taking your cholesterol/blood pressure medication means: taking the entire dose you and your doctor agreed was right for you without forgetting, missing, skipping, or adjusting your dose on your own; and contacting your doctor if you have questions, concerns, or are unsure about if you need to stay on the medication [33]”. Participant responses were coded to a binary outcome of pre-action (not meeting criteria) or action (meeting criteria).

#### 2.4.3. Season

Temperature and hours of sunlight in Alaska can determine food access and food availability. Based on Nome’s calendar and in consultation with advisors in the region, the season when participants completed the nutrition measure was coded as winter (October–April) or other season (May–September).

#### 2.4.4. Community Connectedness

Community connectedness is a predominant feature of Alaska Native traditional culture and shown to be associated with traditional food intake [9,10]. This was measured at baseline with a MacArthur ladder question: “How connected do you feel to the larger community? On a scale from 1 to 10, with 1 representing the lowest amount of connectedness and 10 representing the greatest amount of connectedness [41]”.

### 2.5. Data Management and Analyses

#### 2.5.1. Missing Data

Figure 1, the CONSORT diagram, shows survey and counseling session completion at each timepoint. Retention for the full sample at the 18-month assessment was 79%.

For the 34-item nutrition measure, we required ≥80% of the items answered (i.e., at least 28), resulting in two survey results being excluded from analyses. For participants with at least 28 of 34 of the food items, any missing values were imputed based on the mean of the sample’s responses for that food at that assessment timepoint. Two participants who did not provide information about medication use at baseline and said they were not prescribed medication at each subsequent assessment were not included in the medication adherence analyses.

Survey completion did not differ between groups at any timepoint (chi-squared test *p*-values all >0.05). We performed regression analyses to check for association between survey completion and age, community connectedness, winter, and baseline primary outcomes. Age was significantly associated with survey completion in the nutrition analysis (not for medication adherence) and was included as a covariate in the final analysis. We also observed a significant association between nutrition survey completion and baseline traditional heart-healthy food consumption, but this association was no longer significant when age was added to the model.

Of note, there was a shorter window of time to complete the 3-month assessment relative to the other assessment time points; therefore, this time point had the biggest attrition rate. Most issues with internet connectivity experienced in some locations did not affect counseling, which could be performed by phone instead of the computer.

#### 2.5.2. Data Analyses

To determine the effect of condition over the four follow-up periods on the nutrition outcomes, we ran mixed-effects models with participant ID as a random effect, and season and community connectedness as covariates due to their possible influence on traditional eating. The community research board noted that younger community members are less likely to uphold the traditional food practices of the elders; therefore, we first looked for three-way interactions between condition, time, and age. If the three-way interaction was not significant, we only included age as a covariate. The nutrition outcomes appeared to be somewhat nonnormally distributed (Shapiro–Wilk test *p* < 0.01), so as a sensitivity analysis, we ran identical mixed-effects model analyses on the same outcomes with a square root transformation and observed qualitatively similar results.

To determine the effect of condition across the four follow-up periods on the medication adherence outcomes, we ran generalized mixed-effects models with a binomial distribution fit with maximum likelihood, with ID as the random effect and age as a covariate. Only participants prescribed medications were included in modeling of these outcomes, and the analysis of stage of change outcomes was limited to those participants prescribed cholesterol-lowering, blood-pressure-lowering, or both medications.

Plots for the estimates at each time point were made with the R package emmeans. The study was powered to detect changes in tobacco use, and this paper reports on the study arm that received diet and medication adherence counseling. Our sample size did not provide sufficient power to perform alpha corrections for the multiple outcome measures in this multiple risk behavior trial.

## 3. Results

### 3.1. Participant Characteristics

Table 2 shows the sample baseline characteristics by study condition and for the full sample. The sample was nearly balanced by gender and ranged in age from 19 to 81 years (age mean = 46.3, SD = 14.0; median = 47; interquartile range = 24). Most participants had graduated high school, and 23% resided in Nome. Participant demographic characteristics were similar by study condition. For nutrition measures at baseline, the average proportion of heart-healthy foods was about 41%, and that for traditional heart-healthy foods was 18%, comparable by condition. The bottom of Table 2 shows, for participants who were prescribed heart medication at baseline, measures of medication adherence, which were comparable by condition, and higher for recent (i.e., taking medication as prescribed yesterday, 75%) than typical (i.e., regularly taking their medications, 50%).

### 3.2. Nutrition Outcomes

For the heart-healthy foods ratio, there was no higher-level interaction for condition by time by age, so age was included only as a covariate. The mixed-effects model analysis suggested a statistically significant increase in the heart-healthy foods ratio for participants randomized to the nutrition intervention at 6 months (beta = 0.060, *p* = 0.014) but at no other time point. Age was a significant covariate (beta = 0.0034, *p* < 0.0001). Figure 1 shows the means and standard errors for each condition at each time interval and sample size. Table 3 shows the average servings per week of heart-healthy and unhealthy foods at each time point by condition.

**Figure 2:**
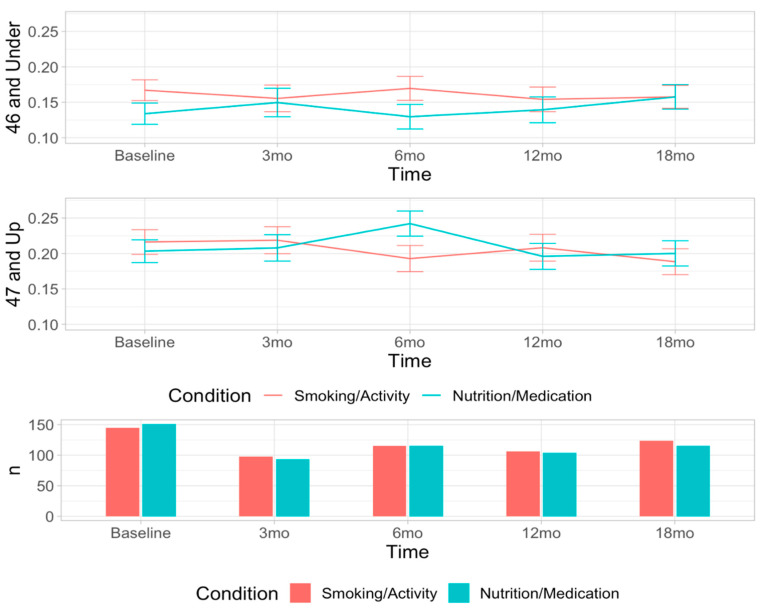
Heart-healthy foods ratio by condition at each time point. Means and standard deviations for the ratio of heart-healthy food servings reported/total servings of foods reported are depicted for each condition at each time point. The sample size of those who filled out the survey at each time point are depicted in the bar chart below the graph.

**Figure 3:**
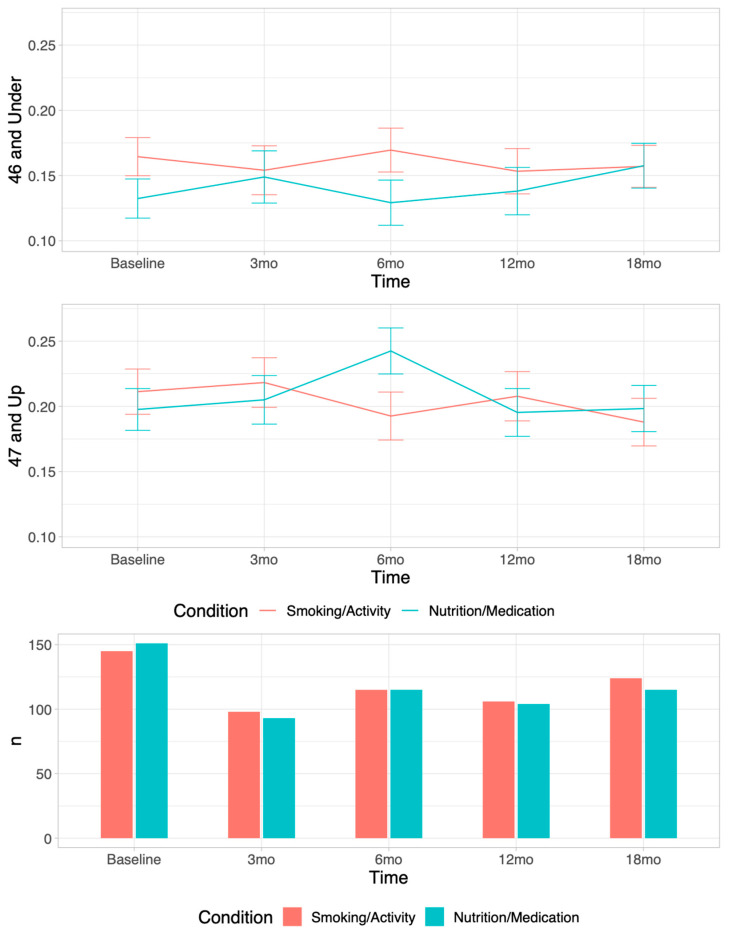
Traditional heart-healthy foods ratio by condition at each time point by median age split at 47 years. Means and standard deviations for the ratio of *traditional* heart-healthy food servings reported/total servings of foods reported are depicted for each condition at each time point; graphs split on median age of 47 years.

For the traditional heart-healthy foods ratio, there was a higher-order significant interaction effect for condition × time × age at 6 months (beta = 0.0036, *p* = 0.013). To better understand treatment response by age, we ran a median split of the sample’s age (47 years old) and depicted the ratio of traditional heart-healthy over time by condition. As shown in Figure 2, participants 47+ years old receiving the nutrition intervention had a significant group difference at 6 months (beta = 0.062, *p* = 0.031) that then waned, while for participants 46 and under, the intervention had no impact. Figure 3 displays the means and standard deviations for the traditional heart-healthy food servings over time within age group by condition.

### 3.3. Medication Adherence

Medication adherence was modeled only with data from participants who were on heart medications (Nutrition/Med n = 72, Tobacco/Physical Activity n = 68). There were no significant group differences on recent or typical medication adherence. There was a significant group difference in the secondary outcome of stage of change for blood pressure medication adherence at 6 months driven by the comparison condition (effect estimate = 1.78, *p* = 0.030). Of note, all four adherence measures started out relatively high at baseline, and the one significant finding was a temporary change in the comparison condition. Outcome graphs and sample sizes for each medication by condition are in the Supplementary Materials.

## 4. Discussion

Delivering innovative, culturally targeted, remotely deliverable, and effective interventions for CVD prevention is an important and needed endeavor. HEALTHH was a multi-behavioral intervention, delivered via telemedicine to Alaska Native adults in their community clinics. Intervention effects were significant for some healthy eating proxies midway through the study period, though the effect size was small. The study was novel in delivering CVD prevention counseling via telemedicine to remote regions of Alaska; tailoring the interventions for relevance to the region and Alaska Native culture; and comparing two distinct, multiple risk behavior interventions so that all participants received counseling on CVD risks.

While, overall, we found limited evidence that the intervention improved nutrition and medication adherence, there was indication of a possible treatment effect at 6 months on heart-healthy foods. Additionally, age related to both study participation and diet, particularly the ratio of traditional heart-healthy foods to other foods. At baseline, older (47 and up) participants reported eating a greater proportion of traditional heart-healthy foods, and during the study, older participants in the nutrition intervention group showed a relative boost in traditional heart-healthy foods at 6 months compared to the comparison group. As reported by members of the community and in published literature, more recent generations tend to have a diet consisting of fewer traditional foods than older generations whether due to globalization, climate change, or other reasons [42]. Younger participants appear more prone to select readily available, highly processed Western foods [9,10]. Targeting the cultural diet intervention to younger adults, with more explicit instructions on how to procure and prepare more traditional foods, may be warranted for future interventions. Additionally, earlier intervention with youth (under 18) may be beneficial. For example, a successful pilot intervention with middle and high school Alaska Native students focused on improving attitudes around traditional foods and increased fish intake and diet quality [7].

Of note, a majority of participants in both conditions reported recent adherence to heart medications at baseline (~77%), and both groups appeared to improve over time in typical medication adherence. This is a positive outcome that could, in part, be explained by both groups simply being asked about their adherence to medication at each study measurement time point and by study participants having access to and coverage for heart medications in their health care system.

As noted earlier, a study strength was the use of telemedicine to deliver CVD prevention counseling in a remote region of Alaska. The telemedicine component was an important means of providing access to health coaching in remote regions in Alaska. Previous reporting of telehealth counseling satisfaction in a subset of the sample was high overall, with mean scores greater than 8 out of a possible score of 10 across treatment conditions, time, community, and gender [43]. Another strength, particularly for the nutrition intervention, was the engagement of Alaska Native men and women in culturally targeted interventions. The nutrition messaging was attentive to seasonal variability and affordability challenges in access to fresh produce and to traditional food sharing and procurement. Having a culturally targeted nutrition intervention with measures that attended to geographic accessibility and cultural values make this an important contribution to the larger field of nutrition studies promoting heart-healthy eating. A third strength is that the design compared two active interventions and multiple risk behaviors. While this leads to multiple primary outcomes statistically, having an active control and providing counseling on multiple health behaviors optimizes potential impact and study retention for all participants.

Study retention of 79% at 18 months exceeded the CDC guidance of 70% retention or higher [44]. Due to various and sometimes unspecified reasons (e.g., internet connectivity issues, assessment fatigue, participant not knowing an answer), there were missed assessments and missed counseling sessions, which weakens the dose of the intervention and statistical power to detect treatment effects. The study design minimized participant burden by having surveys at the same study touchpoints as counseling sessions, but this does lead to a correlation between measurement and counseling.

A study limitation was the reliance on self-reported outcome measures. The desire to please may have led to response bias in the reported medication adherence and food choices. Additionally, because the diet measure was a short list of foods, we cannot determine if the non-heart-healthy foods that were decreased were replaced by other unhealthy foods that were not on the list (e.g., swapping out Crisco for bacon lard). Short food frequency questionnaires have been shown to be valid for monitoring changes in food patterns over time [45]. A more complete diet assessment would include multiple 24 hour recalls, though these are time-intensive, and one study on women found this method to be less valid than a semi-quantitative food frequency questionnaire [46]. Further, for this intervention, we prioritized the increase in traditional heart-healthy foods, specifically a subset of foods included in the cookbook and reviewed by team members of Alaska Native heritage and members of the community board; therefore, we adapted the questionnaire measure to ask about our specific foods of interest instead of a free recall or an exhaustive list of all possible foods eaten. The measure may not be sensitive enough to detect small changes in diet, even among those foods assessed. Finally, we chose to do a median split on age based on the sample to look at the traditional food intake. A more optimal age split by generation or a different age cut-point may be warranted for future work and could yield different outcomes.

## 5. Conclusions

Heart disease contributes to significant morbidity and mortality in Alaska Native people [1,2]. Given unique differences in land, weather, community living, cultural values, and lifestyles in the Norton Sound region of Alaska, this study developed and deployed a culturally targeted, multi-behavioral, telehealth intervention that both addresses and promotes these unique features to promote heart health. While more touchpoints may be necessary to impart lasting change, the study is novel in testing telemedicine-delivered, culturally targeted nutrition and medication adherence interventions in a remote region of Alaska. This study established the feasibility of the telehealth modality in reaching remote populations, positive acceptance of the culturally tailored intervention, and a format for intervening on multiple CVD risk behaviors at once while maintaining a randomized design. Future work can incorporate these key features in programs to advance the health of Alaska Native people.

## Figures and Tables

**Table 1:** Comparison of intervention components.

Treatment Component	Condition 1: Smoking Cessation + Physical Activity	Condition 2: Nutrition and Medication Adherence
Manual ^1^	Provided at baseline	Provided at baseline
30-min Counseling ^1^ Telehealth, computerized Tailored to condition and participant	Baseline, 3-mo, 6-mo, 12-mo	Baseline, 3-mo, 6-mo, 12-mo
Prochange report (mailed)	After Baseline, 3-mo, 6-mo, 12-mo	After Baseline, 3-mo, 6-mo, 12-mo
Swag	Provided at baseline. Items: Pedometer; 12-weeks of NRT	Provided at baseline. Items: Cookbook; Medication bag

^1^ Note: The manual and each stage of change treatment were based on Prochaska’s TTM model [30,33].

**Table 2:** HEALTHH Study Participant Baseline Characteristics (N = 299).

	Nutrition + Medication Adherence (N = 151)	Tobacco + Physical Activity (N = 148)	Full Sample (N = 299)
	M (SD)/N (%)	M (SD)/N (%)	M (SD)/N (%)
**Age** (M/SD)	47.0 (13.4)	45.5 (14.7)	46.3 (14.0)
**Gender** (N/%)			
Male	77 (51.0%)	77 (52.0%)	154 (51.5%)
Female	74 (49.0%)	71 (48.0%)	145 (48.5%)
**Highest Education Level** (N/%)			
Elementary/some high school	38 (25.2%)	23 (15.5%)	61 (20.4%)
High school graduate	83 (55.0%)	95 (64.2%)	178 (59.5%)
Some college	26 (17.2%)	26 (17.2%)	52 (17.4%)
College degree or higher	3 (2.0%)	4 (2.7%)	7 (2.3%)
**Community Connectedness** (M/SD)	5.7 (2.5)	5.6 (2.6)	5.3 (2.1)
**Residence** (N/%)			
Nome	31 (20.5%)	38 (25.7%)	69 (23.1%)
Another Norton Sound community	120 (79.5%)	110 (74.4%)	230 (76.9%)
**Nutrition Measures** (M/SD)			
Heart-healthy foods ratio (%)	40% (19)	42% (20)	41% (20)
Traditional healthy foods ratio ^1^	17% (13)	19% (15)	18% (14)
Total servings heart-healthy foods ^2^	19.9 (15.2)	21.5(20.4)	20.7 (18.0)
Total servings traditional heart-healthy foods	8.5 (9.3)	9.5 (11.0)	9.0 (10.1)
Total servings unhealthy foods	27.9 (18.1)	27.7 (21.8)	27.8 (20.0)
**Medication Adherence** ^3^			
Recent medication adherent	55/71 (77%)	49/67 (73%)	104/138 (75%)
Typical medication adherent	36/72 (50%)	33/67 (49%)	69/139 (50%)
Action ^4^: Blood pressure medication	44/57 (77%)	48/67 (72%)	92/124 (74%)
Action ^4^: Cholesterol medication	33/40 (83%)	24/38 (63%)	57/78 (73%)

^1^ Ratio indicates the number of heart-healthy food servings reported/the number of total food servings reported and represents the percentage of the surveyed foods that come from heart-healthy foods. ^2^ Total servings indicate the total number of reported servings per week for the food category. ^3^ Among those prescribed heart medications. ^4^ Action indicates in either Action or Maintenance stage for medication adherence in the Stages of Change model [33].

**Table 3:** Unhealthy foods, heart-healthy foods, and traditional heart-healthy foods servings by condition over time.

		M (SD) Servings per Week			
Group	Baseline	3-mo	6-mo	12-mo	18-mo
**Nutrition/Medication Adherence**					
Unhealthy foods	27.9	27.8	26.5	24.4	26.0
	(18.1)	(16.6)	(17.1)	(17.7)	(21.2)
Heart healthy foods	19.9	20.2	21.9	19.2	20.2
	(15.2)	(13.0)	(15.5)	(15.0)	(16.5)
Traditional heart-healthy foods, age ≤ 46 years ^1^	5.5	7.2	5.9	5.6	6.9
	(6.3)	(6.4)	(4.8)	(4.9)	(5.9)
Traditional heart-healthy foods, age ≥ 47 years ^1^	11.1	9.5	12.0	9.7	9.8
	(10.8)	(8.8)	(9.6)	(9.7)	(9.9)
**Tobacco/Physical Activity**					
Unhealthy foods	27.7	26.2	26.9	25.9	26.0
	(21.8)	(17.0)	(16.9)	(16.2)	(13.4)
Heart healthy foods	21.5	20.1	20.9	20.8	20.7
	(20.4)	(13.9)	(16.3)	(15.8)	(14.0)
Traditional heart-healthy foods, age ≤ 46 years ^1^	8.3	6.7	7.9	7.8	7.9
	(8.4)	(5.6)	(7.2)	(8.3)	(8.3)
Traditional heart-healthy foods, age ≥ 47 years ^1^	10.8	10.0	9.8	8.8	7.1
	(13.1)	(8.2)	(8.8)	(7.4)	(5.6)

^1^ Traditional heart-healthy foods split by median age over time.

## Data Availability

Access to the data is restricted for tribal protections.

## Supplementary Materials

The following supporting information can be downloaded at: https://www.mdpi.com/article/10.3390/ijerph19169885/s1.
