# Supplementary material for: Traditional Heart-Healthy Diet and Medication Adherence in the Norton Sound Region: An 18-Month Telehealth Intervention
Source: Int J Environ Res Public Health. 2022 Aug 11;19(16):9885. doi: 10.3390/ijerph19169885 (PMC9408057; doi:10.3390/ijerph19169885)
Supplement: Supplementary file 1 [file ijerph-19-09885-s001.zip › ijerph-1849185-supplementary.pdf]

Think about all the foods you ate over the past 7 days as part of a meal or as a snack. I am going to ask how often you ate each of the following food items. Response options are "did not eat it this week," "once this week," "2 to 3 times this week," "4 or 6 times this week," "once or twice each day," and "more than twice each day." Please count all foods. For example, if you ate oatmeal with nuts and milk, please report the oatmeal, the nuts, and the milk separately.

In the past 7 days how often did you drink or eat...

	**Did not eat it this week**	**Once this week**	**2-3 times this week**	**4-6 times this week**	**Once or Twice each day**	**More than twice each day**	**Refused**
**Sweetened fruit drinks**	0	1	2	3	4	5	-
**Fruit Juice**	0	1	2	3	4	5	-
**Soda**	0	1	2	3	4	5	-
**Low fat or nonfat milk**	0	1	2	3	4	5	-
**Fruit canned in syrup**	0	1	2	3	4	5	-
**Applesauce with sugar added**	0	1	2	3	4	5	-
**Wild berries / Salmon berries (fresh or frozen)**	0	1	2	3	4	5	-
**Apples, bananas, or other whole fresh fruit**	0	1	2	3	4	5	-
**French fries or chips**	0	1	2	3	4	5	-
**Celery, Eskimo spinach, traditional greens**	0	1	2	3	4	5	-
**Canned Beets**	0	1	2	3	4	5	-
**Carrots**	0	1	2	3	4	5	-
**Lettuce**	0	1	2	3	4	5	-
**Sugar cereals**	0	1	2	3	4	5	-
**Hot pockets, pizza**	0	1	2	3	4	5	-
**Donuts, fried bread, or Assaliaq (donut)**	0	1	2	3	4	5	-
**Assaliaq but substitute seal oil**	0	1	2	3	4	5	-
**Ramen noodles**	0	1	2	3	4	5	-
**Oatmeal or mush**	0	1	2	3	4	5	-

**Spam**	0	1	2	3	4	5	-
**Crisco**	0	1	2	3	4	5	-
**Seal or whale oil**	0	1	2	3	4	5	-
**Nuts and seeds**	0	1	2	3	4	5	-
**Fried salmon**	0	1	2	3	4	5	-
**Whale skin and fat, Muktuk**	0	1	2	3	4	5	-
**Moose, caribou, reindeer**	0	1	2	3	4	5	-
**Fish and succotash**	0	1	2	3	4	5	-
**Dried / baked / broiled salmon / Tupa (fermented salmon)**	0	1	2	3	4	5	-
**Fish soup, walrus soup, moose stew, caribou stew**	0	1	2	3	4	5	-
**Lowfat or nonfat yogurt**	0	1	2	3	4	5	-
**Cool whip**	0	1	2	3	4	5	-
**Ice cream or Agutuk (Eskimo ice cream)**	0	1	2	3	4	5	-
**Packaged bar (wrapper desert, pastry, chocolate, candy, or energy bar)**	0	1	2	3	4	5	-
**Packaged meal (frozen meal)**	0	1	2	3	4	5	-
